# An Exploratory Investigation of E-Rest: Teletherapy for Chronically Aphasic Speakers

**DOI:** 10.5195/ijt.2016.6191

**Published:** 2016-07-01

**Authors:** MARINA B. RUITER, TONI C.M. RIETVELD, VERA HOSKAM, MARIJN M.A. VAN BEERS

**Affiliations:** 1 SINT MAARTENSKLINIEK, NIJMEGEN, THE NETHERLANDS; 2 CENTRE FOR LANGUAGE STUDIES, FACULTY OF ARTS, RADBOUD UNIVERSITY, NIJMEGEN, THE NETHERLANDS; 3 ICT SERVICE CENTRE (ISC), RADBOUD UNIVERSITY, NIJMEGEN, THE NETHERLANDS

**Keywords:** Agrammatism, Chronic aphasia, e-REST, Telepractice, Teletherapy

## Abstract

Delivering aphasia therapy via telecommunication may provide a means to deliver intensive therapy in a cost-effective way. Teletherapy, remotely-administered (language) treatment, may support the repetitive drill practices that people with chronic aphasia need to perform when learning to compensate for their lasting language difficulties. The use of teletherapy may allow speech and language pathologists (SLPs) to focus in-person sessions more strongly on the generalisation of therapy effects to daily life. This single subject study is an investigation whether a teletherapy application called e-REST meets the criteria of accessibility, user-friendliness, as well as effectiveness. e-REST, the teletherapy version of the Dutch and adapted Reduced Syntax Therapy, teaches chronically aphasic speakers of Dutch who experience difficulties in sentence production to convey their messages in a kind of telegraphic style. The results obtained suggest that it is reasonable to conduct a larger study into the user-friendliness, accessibility, effectiveness, and cost-effectiveness of e-REST.

Aphasia can be defined as an acquired communication disorder caused by brain damage. It is characterised by an impairment of speaking, listening, reading, and/or writing due to a reduction or impairment of language and the non-linguistic cognitive processes that underlie or interact with language (i.e., executive functions). As a result, functional communication skills – that is, the skills which are needed to get the message across both effectively and efficiently in daily life – are diminished. When language skills cannot be completely regained spontaneously or as a result of language therapy, aphasia becomes a chronic condition (Hallowell & Chapey, 2008; McNeil & Pratt, 2001).

There is a growing body of evidence that highly intensive SLP services of a sufficient duration are more effective than less intensive ones (Barthel, Meinzer, Djundja, & Rockstroh, 2008; Basso & Macis, 2011; Brady, Kelly, Godwin, & Enderby, 2012; Brindley, Copeland, Demain, & Martyn, 1989; Code, Torney, Gildea-Howardine, & Willmes, 2010). However, the resources required to deliver highly intensive SLP services become increasingly insufficient (Ministerie van Volksgezondheid, 2012; Theodoros, 2012). When resources are lacking to deliver highly intensive aphasia treatment in an in-person manner over a sufficient period of time, SLPs may be able to treat chronic aphasia both intensively and cost efficiently via information and communication technology (ICT), also known as *telerehabilitation* (Beijer & Rietveld, 2015; Cherney & Van Vuuren, 2012; Code, 2012; Parmanto & Saptono, 2009; Rogante, Grigioni, Cordella, & Giacomozzi, 2010).

Parmanto and Saptono (2009) have distinguished various types of telerehabilitation; one of these is *teletherapy*, which will be the focus of this article. Teletherapy refers to the situation in which a patient or client carries out therapeutic activities in the home setting while the therapist manages the course of therapy remotely, via the Internet. Teletherapy may contribute to a cost reduction of aphasia therapy as it enables persons with aphasia to complete at least a part of a therapy programme without the therapist being present (Code & Petheram, 2011; Mortley, Wade, & Enderby, 2004).

Teletherapy may be specifically useful for the drill and practice aspects of compensation therapy, which is typically conducted in the chronic phase. With compensation therapy, persons with aphasia are taught to use a part of their intact verbal or non-verbal communication functions to circumvent the impaired language component. For example, chronic sentence production difficulties (i.e., expressive agrammatism) may be compensated by the continuous use of reduced utterances called *ellipses* (Kolk, 2006; Progovac, 2006). Ellipses demonstrate resemblance to the telegraphic style that people without aphasia also use from time to time (e.g., *Anyone hungry? Sandwiches on the table!*). In the study of Ruiter et al. (Ruiter, Kolk, & Rietveld, 2010; Ruiter, Kolk, Rietveld, & Feddema, 2013), a face-to-face therapy called the Dutch and adapted Reduced Syntax Therapy (REST; for the original German version of the REST see Schlenck, Schlenck, & Springer, 1995) significantly enhanced elliptical style in Dutch-speaking persons with chronic agrammatism. The continuous use of ellipses allowed these speakers to improve functional communication due to an enhancement of verbal efficiency.

Teletherapy may partly replace the in-person therapy sessions allocated to the drill and practice of the new compensatory behaviour in order to achieve a clinically significant enhancement of a patient’s behaviour; however, its delivery should be both effective and efficient. Mortley, Wade, and Enderby (2004) argue that the applications and technologies involved should therefore fulfil at least the following features. First, teletherapy should be accessible and easy to use by people with aphasia. Second, therapists should be able to monitor their clients’ performance from a distance and to adjust the complexity of the training items via remote access when required. Moreover, there should be evidence for the effectiveness of the therapy program delivered remotely. Lastly, the amount of practice time in the teletherapy application should outweigh the time required for the therapist to manage their clients’ progress, involving actions like scoring responses and assigning new therapy exercises. The latter feature implies that persons with aphasia should be able to run through the therapy exercises by themselves, at least for the greater part (Cherney & van Vuuren, 2012).

In summary, on the one hand, teletherapy has the potential to deliver the drill and practice aspects of compensation therapy both intensively and cost efficiently. Conversely, there may be various barriers to overcome before it can be used in clinical practice. The aim of the current single subject study is to explore whether the potential promises of a teletherapy version of the Dutch and adapted Reduced Syntax Therapy (Ruiter et al., 2010) - henceforward referred to as e-REST (to be described in detail below) - outweigh its potential limitations. We do so by investigating whether e-REST meets the criteria of Mortley et al. (2004), among which are accessibility, user-friendliness, and effectiveness.

## METHOD

### DESIGN AND PARTICIPANT CHARACTERISTICS

In this single-case intervention study, e-REST was administered following a protocolised training procedure (Ruiter, 2008) for a total of 22 hours over a period of 16 weeks to a Dutch-speaking adult with chronic expressive agrammatism (*N* = 1). Both before (T1) and after e-REST (T2), the effect of the intervention on grammatical output and functional communication was investigated with a Picture Description Task (PDT, to be described below), which is built into the eREST application and routinely administered in clinical practice as a clinimetric analysis of this intervention. The current single subject study was approved by the scientific review committee of the Sint Maartenskliniek rehabilitation centre, from which the participant received an interdisciplinary rehabilitation programme, including language therapy for his chronic aphasia. Before being enrolled in e-REST, participant JR (fictitious initials) signed an informed consent form indicating that he approved of his spoken responses being anonymously stored on the central server and being used for scientific purposes.

Participant JR, a 71 years old right-handed male who had received an intermediate vocational education level, was 7 months post onset of a left middle cerebral artery infarct at the pre-therapy measurements (T1). At T1, he produced agrammatic speech output and based on his scores on the Dutch Aachen Aphasia Test (AAT; Graetz, de Bleser, & Willmes, 1992), he was diagnosed with Broca’s aphasia of an average severity (z = .05). In addition to these aphasic symptoms, he also demonstrated moderate apraxia of speech. As a result, his speech output was rated as 80% intelligible. JR was included as he fulfilled both the linguistic criteria for e-REST and was familiar with computers premorbidly.

### MATERIALS

#### E-REST

In line with the in-person version of the Dutch and adapted REST (Ruiter et al., 2010, 2013), e-REST consisted of 525 therapy items, which were divided over ten levels. In e-REST, therapy was asynchronously administered via the Internet. That is, although the SLP and JR were both engaged in e-REST, they did not necessarily have to do so at the same time. Both had access to a central server which contained both the audio files of the ellipses targeted in therapy (in .mp3 format) and the uploaded audio files (in .flv format, that is as Flash video files) of the speech that JR produced to the therapy and test items. When uploaded, each .flv file was automatically converted to an ordinary .mp3 format as a means to compress the audio recordings. The web-based therapy programme had two interfaces: one for the aphasic client (front end) and one for the SLP (back end).^a^

#### USER INTERFACE

**Figure 1:**
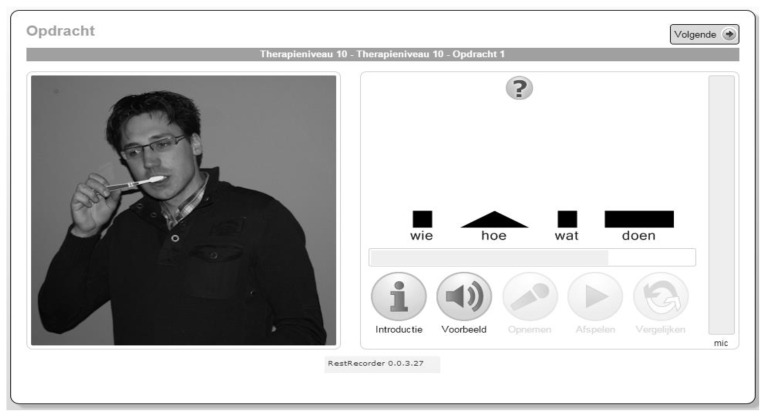
User interface of the first item of the tenth therapy level of e-REST when enrolled in the first therapy cycle. Target elliptical utterance: *Paul zonder tandpasta tanden poetsen*, ‘Paul brushing teeth without toothpaste’. For an explanation of the buttons depicted in the interface see the text.

Figure 1 displays the interface of the first therapy item in the first cycle of the tenth therapy level (target ellipsis was *Paul zonder tandpasta tanden poetsen, ‘*Paul brushing teeth without toothpaste’). A picture that had to be described was displayed on the left side of the screen. The syntactic structure of the ellipsis was visualised on the middle of the computer screen. Each constituent was depicted by a different visual symbol (a circle, a square, etc.). Together with these graphic cues, the structure of the ellipsis was also modelled by written cues. The user interface also contained five buttons, which were located below the cue bar. In Figure 1, only the first two buttons, the *Introductie*, ‘Introduction’ and the *Voorbeeld*, ‘Example’ buttons were active (in grey colour). The last three buttons – the *Opnemen*, ‘Record’, *Afspelen*, ‘Play’, and *Vergelijken*, ‘Compare’ buttons – were inactive.

#### THERAPIST INTERFACE

**Figure 2:**
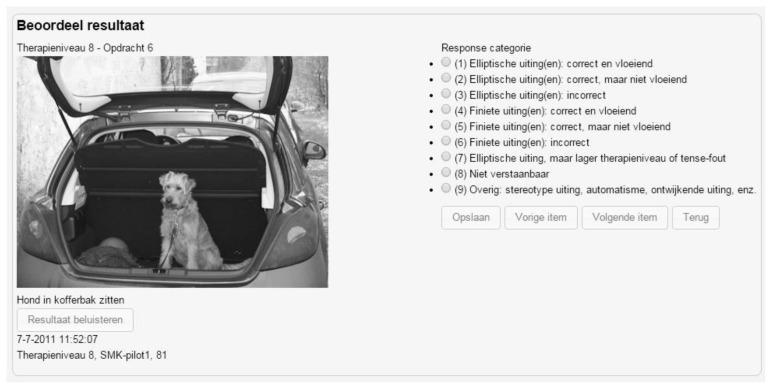
Therapist interface of the sixth item (target utterance: *Hond in kofferbak zitten*, ‘Dog sitting in trunk of the car’) when enrolled in the second cycle of the eight therapy level (for an explanation see text).

Figure 2 presents the therapist interface. By clicking on the *Resultaat beluisteren*, ‘Hear client’s response’ button, the SLP could listen to the response that JR had produced to each item. The SLP then scored the response digitally by clicking one of the nine response categories. In these categories the most prominent subdivision was one between ellipses and sentences (in Dutch *elliptische uitingen* categories 1 to 3] and *finiete uitingen* [categories 4 to 6] respectively, for further details see Ruiter (2008).

#### HARDWARE

Both the SLP and JR had access to a PC that had a sound card and (at least) Windows XP as an operating system. Both computers were supplied with an Internet connection and were equipped with Flash Player, tenth version (or higher) as well as one of the following browsers with the given version numbers (or higher): Internet Explorer 7.0, Mozilla Firefox 3.0, or Google Chrome. Further requirements consisted of the ability to capture high-resolution (1024 × 768 pixels) and TCP port 1935 of the firewall to be opened. A standard mouse and a Sennheiser PC 131 headset were used in order to allow listening to the audio examples of target utterances as well as to allow recording of the spoken responses via the headset’s microphone in order to upload it to the central server.

### TREATMENT PROCEDURE

In order to be able to qualitatively analyse the user-friendliness and accessibility, JR accessed e-REST via a computer located at the rehabilitation centre. The same protocol that was employed to evaluate the efficacy of the in-person version of REST (Ruiter et al., 2010) was used in the current study; however, there was a difference. With the start of each new therapy level in e-REST (i.e., first cycle), it was not possible for JR to record his spoken response after clicking the *Voorbeeld*, ‘Example’ button, as this would give an invalid reflection of his skill to produce ellipses independently. Instead, once all items had been listened to, the same items would be presented for as second time (i.e., second cycle). In this second cycle, the example could only be accessed following the recording of a response (without being able to make a new recording). When all items of the second cycle of that therapy level had been completed an email was automatically sent to the SLP, who could then score each response digitally. Subsequently, a new cycle of the same therapy, a new (higher) level, or an intervening test would be presented (for details see Ruiter (2008)).

### OUTCOME MEASURES

Both before (T1) and after enrolling into e-REST (T2) a Picture Description Task (PDT; Ruiter, 2008) was administered. The PDT consisted of 40 pictures of everyday life activities and was implemented in the e-REST application.^b^ The spoken responses to the PDT were analysed as described in Ruiter et al. (2010; 2013), which yielded the following outcome measures:

- The *percentage of Words produced In Ellipses*: parameter *%WIE,* in order to indicate the effect of e-REST on grammatical output.
- *Verbal effectiveness.* The percentage of *Essential Content Units (ECUs),* which is the percentage of essential information units that a listener needs to understand the message (Yorkston & Beukelman, 1980).
- *Verbal efficiency.* The (average) *Number of Essential Content Units per Minute* (i.e., ECU/min).

### STATISTICAL ANALYSES

The Likelihood Ratio option of Chi-Square (*α* = .05, one-tailed) was calculated to investigate change in grammatical output and verbal effectiveness over time (T1 to T2) and the contingency coefficient, *C*, which ranges from 0 to 1, in order to indicate the strength of these effects. We used the Likelihood Ratio test (an exact test) instead of the Chi-Square test, as some of the expected values of the cell frequencies were less than 5. In addition, we used Welch’ *t-*test and Wilcoxon’s signed-rank test (*α* = .05, one-tailed) to investigate change in verbal efficiency over time and Cohen’s *d* to indicate the strength of the effects on verbal efficiency. The Holm method (Holm, 1979) was used to control for inflation of the Type I error inherent to multiple testing.

## RESULTS

### ACCESSIBILITY AND USER-FRIENDLINESS

The e-REST teletherapy application could be used independently for the greater part by JR once the SLP had initialised the application on the computer, selected the therapy item with which to proceed, and made sure that the headset microphone worked properly. Although some difficulties with the recording of the responses was observed, the number of unrecorded responses never exceeded 5*%*.

### EFFECTIVENESS

#### ABILITY TO USE ELLIPTICAL STYLE REGULARLY ON TRAINED MATERIAL

Six of the ten therapy levels were completed in 22 e-REST sessions of an hour each. Elliptical style was produced on at least 90% of the (trained) pictures for these levels.

#### EFFECTS OF E-REST ON GRAMMATICAL OUTPUT PRODUCED ON UNTRAINED MATERIAL

With respect to the effect of e-REST on grammatical output a first outcome was that significantly more ellipses were produced after therapy. More specifically, the percentage of words produced in ellipses (*%WIE*) on the PDT significantly increased from 3,1*%* at T1 to 41,5*%* at T2, *LR* (1) = 88.141, *p* = 0.000, which represents quite a large effect (*C* = 0.442).

**Figure 3:**
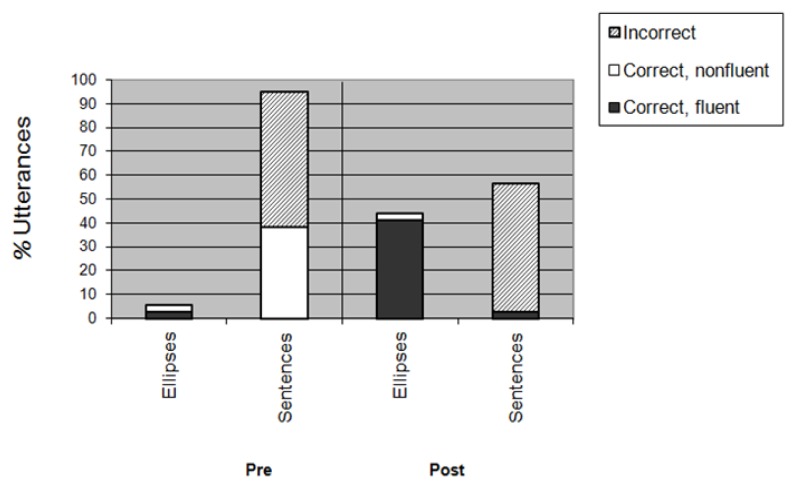
Type of ellipses and sentences produced on the pre-therapy (T1) and post-therapy (T2) Picture Description Task (PDT: for an explanation see text).

Secondly, fluent ellipses occurred significantly more often after than before therapy. Figure 3 displays the type of ellipses and sentences produced on the pre- and post-therapy PDT and illustrates that significantly more grammatically well-formed and fluent ellipses were produced after therapy (41.03*%*) in comparison to the pre-therapy measurement (2.56*%*), *LR* (1) = 19.687, *p* = 0.000, *C* = 0.422. In contrast, significantly less well-formed but non-fluently produced sentences occurred after therapy (0.00*%*) when compared to the pre-therapy measurement (35.90*%*), *LR* (1) = 22.496, *p* = 0.000, *C* = 0.424.

Thirdly, the percentage of incorrect sentences did not diminish significantly from T1 (58.97*%*) to T2 (53.85*%*), *LR* (1) = 0.209, *ns, C* = 0.052. Neither did the percentage of fluently and correctly produced sentences significantly increase over time (2.56*%* at T1 as well as 2.56*%* at T2, *LR* (1) = 0.000, *ns, C =* 0*.*000)*.* In relating the second and third mentioned findings, it seems that the production of fluent and correct ellipses was enhanced – at least for the major part – at the expense of the effortful production of well-formed sentences.

#### EFFECTS OF E-REST ON FUNCTIONAL COMMUNICATION

The average number of ECUs produced per minute (ECUs/min) increased from 10.10 at T1 to 27.58 at T2. Although the scores were obtained in a matched samples design, a Welch’ *t* test for independent samples was used. There were two reasons for not applying a *t* test for dependent samples: 1) the scores obtained at the two moments in time were not correlated (*r* = −0.065, *p* = 0.697); in that case a *t* test for independent samples is recommended by Wilcox (2012), and 2) the assumption of additivity was not warranted: (F(1,38) of Tukey’s test for additivity was 7.46, *p*= 0.010). The Wilcoxon-signed rank test was not an alternative either as the difference scores were not symmetric (Munzel, 1999). Welch’ t test for independent samples was *t*(69.57) = −12.22, *p* = 0.000 (two-sided), *d* = 2.77; the paired *t* test was *t*(37) = −11.915, *p* = 0.000, *d* = 2.64. Thus, verbal efficiency improved, while the listener was still provided with the same amount of essential information needed to understand the message. That is, the percentage of ECUs produced was 85.60*%* at T1 and 80.65*%* at T2, *LR* (1) = 1.093, *ns, C* = 0.066.

#### TIME REQUIRED BY THE SLP TO MANAGE PROGRESS

Averaged over 40 items, it took about 45 minutes for JR to complete this number of items and 15 to 20 minutes for the trained SLP to score them.

## DISCUSSION

In order to obtain a first indication of the usefulness of e-REST in enhancing elliptical style and consequently improving verbal efficiency, we investigated whether the teletherapy application e-REST satisfies the criteria of Mortley et al. (2004). Although the results obtained have to be interpreted with some caution as the study may not have been optimally controlled (i.e., no control for spontaneous recovery and small speech samples c), e-REST seems to meet most criteria.

In relation to their first criterion - the accessibility and user-friendliness of e-REST - it was observed that JR could run through the therapy items with little help. Help should not necessarily be provided by the SLP, but may be offered at home by a trained significant other or volunteer. It would also be relevant to investigate the prerequisites for (partly) independent use in future research. Premorbid experience with computers may be one of these factors, as the case of JR illustrates.

The second criterion concerns the ability for SLPs to monitor their clients’ performance from a distance and to adjust the complexity of the training items remotely. As we described in the Method section, the therapist interface of e-REST allows the SLP to do both.

Mortley et al. further state that there should be evidence for the effectiveness of the therapy programme run remotely. Although the results obtained with this single subject study have to be interpreted cautiously, they suggest that e-REST allows agrammatic speakers of Dutch to improve their functional communication due to an enhancement of verbal efficiency.

Lastly, it is argued by Mortley et al. that the time required by the SLP to manage the client’s progress, including time needed for scoring, should be considerably less than user’s practice time in the teletherapy application. Although e-REST fulfils this criterion, one could argue that it takes the SLP still relatively much time to score the patients’ exercises manually. Developments in Automatic Speech Recognition (ASR) technology might offer a means to automate the scoring procedure, which in turn may improve the cost efficiency of e-REST. ASR would also allow automatic feedback to the aphasic user, which would further improve user accessibility and friendliness. Research in computer assisted language learning (CALL) and speech therapy has shown that it is feasible to employ ASR to analyse the speech produced by language learners or dysarthric patients, identify possible errors, and provide feedback on those errors to help learners and patients improve their speech production (Beijer & Rietveld, 2011; Penning de Vries, Bodnar, Cucchiarini, Strik, & Van Hout, 2013; Sanders, Ruiter, Beijer, & Strik, 2002; Strik, 2012). Research is needed to determine in which conditions employing ASR for the abovementioned purposes is feasible.

In summary, although the results obtained with this single subject study have to be interpreted cautiously, e-REST seems to have the potential to enhance elliptical style and functional communication in chronically agrammatic speakers of Dutch. Therefore a study on a larger scale into the user-friendliness and effectiveness of e-REST is advisable (Currell, Urquhart, Wainwright, & Lewis, 2000). Future research should also focus on the cost-effectiveness, users’ satisfaction, and the long-term maintenance effects of e-REST.

## NOTES

aAlthough not used in the current study, an e-learning website has been developed which offers significant others of the aphasic client information on the method and procedures used in the e-REST application. SLPs can also access this website for background information and technical support. The site also contains information on the scoring procedures and procedures for assigning therapy levels and intermediate tests to their clients.bThe e-REST version of the PDT is very similar to the PDT that was used in the study of Ruiter et al. (2010, 2013); however, there were very small differences which relate to the fact that new photo material had to be constructed for the e-REST PDT. In total 8 of the 40 photos diverged: seven test items differed with respect to the noun used in the subject position (e.g., *man smoking a cigarette* versus *woman smoking a cigarette*) and one item differed with respect to the noun in the direct object position (e.g., *man feeding the dog* versus *man feeding the cat*).cBoth the T1 and T2 speech samples did not contain at least 300 words as recommended (Boxum, van der Scheer, & Zwaga, 2013; Brookshire & Nicholas, 1994), The speech sample consisted of 229 words at T1 and of 130 words at T2.
